# 3D solid supported inter-polyelectrolyte complexes obtained by the alternate deposition of poly(diallyldimethylammonium chloride) and poly(sodium 4-styrenesulfonate)

**DOI:** 10.3762/bjnano.7.18

**Published:** 2016-02-05

**Authors:** Eduardo Guzmán, Armando Maestro, Sara Llamas, Jesús Álvarez-Rodríguez, Francisco Ortega, Ángel Maroto-Valiente, Ramón G Rubio

**Affiliations:** 1 Departamento de Química Física I-Facultad de Ciencias Químicas, Universidad Complutense de Madrid, Ciudad Universitaria s/n, 28040 Madrid, Spain; 2 Department of Physics - Cavendish Laboratory, University of Cambridge, J. J. Thomson Avenue, CB3 0HE Cambridge, United Kingdom; 3 Istituto per l’Energetica e le Interfasi - U.O.S. Genova, Consiglio Nazionale delle Ricerche, Via De Marini 6, 16149 Genova, Italy; 4 Departamento de Química Inorgánica y Química Técnica - Facultad de Ciencias Universidad Nacional de Educación a Distancia, C/ Senda del Rey 9, 28040 Madrid; 5 Instituto Pluridisciplinar, Universidad Complutense de Madrid Avda. Juan XXIII 1, 28040 Madrid, Spain

**Keywords:** charge compensation, hydration, polyelectrolyte multilayers, stratification, swelling

## Abstract

This work addresses the formation and the internal morphology of polyelectrolyte layers obtained by the layer-by-layer method. A multimodal characterization showed the absence of stratification of the films formed by the alternate deposition of poly(diallyldimethylammonium chloride) and poly(sodium 4-styrenesulfonate). Indeed the final organization might be regarded as three-dimensional solid-supported inter-polyelectrolyte films. The growth mechanism of the multilayers, followed using a quartz crystal microbalance, evidences two different growth trends, which show a dependency on the ionic strength due to its influence onto the polymer conformation. The hydration state does not modify the multilayer growth, but it contributes to the total adsorbed mass of the film. The water associated with the polyelectrolyte films leads to their swelling and plastification. The use of X-ray photoelectron spectroscopy has allowed for deeper insights on the internal structure and composition of the polyelectrolyte multilayers.

## Introduction

The new requirements of science and technology have created an increasing interest for the fabrication of materials with reduced dimensionality for their application in several fields, including optics, electronics, coatings and biomaterials (drug delivery and tissue engineering). In order to create the aforementioned materials, the development of new bottom-up techniques, which allow one to control the properties and structure of the materials at the sub-micrometric scale, has become necessary [1–3]. Among these techniques, the layer-by-layer (LbL) self-assembly has become probably one of the most promising [4–5], due to its high versatility and low costs [6–7]. Furthermore, a very broad range of compounds can be assembled through LbL: synthetic polyelectrolytes, biopolymers – such as peptides, proteins and nucleic acids – colloidal particles, carbon nanotubes, and/or microgels [8–10], which confers to this method an almost unlimited chemical versatility. Even though the method frequently makes use of electrostatic interactions, the multilayers can also be built based on other intermolecular forces, for instance hydrogen bonds, acid–base reactions, covalent cross-linking and host–guest interactions [11–12].

Polyelectrolyte multilayers can be considered an example of non-equilibrium materials, because the corresponding soluble or insoluble complexes are more stable from the thermodynamic point of view [13]. Thus, the structure and properties of the final film are expected to be strongly dependent on the experimental protocol followed for its fabrication. Many variables have strong influence on the final structure of LbL films, hence to know their role during film formation is critical for controlling the structure and physicochemical properties of the films [13]. Among the most relevant variables are the charge density of the molecules, the concentration of the solution used, ionic strength, solvent quality for the molecules, pH, and temperature [13].

In the last years, a lot of theoretical and experimental research effort has been spent to understand the different growth mechanisms that appear during the alternate deposition of the layers, the quantification of the adsorbed amount of material in each adsorption cycle, as well as the developments of technological applications for the manufactured systems [3]. Despite the extensive research, certain aspects that play an important role in the applications for these systems still remain unclear [14]. Among these aspects the internal composition of the multilayers (ionic composition and water content), the internal structure of the films and their mechanical properties are probably the most important [15–19].

This work studies polyelectrolyte multilayers formed by poly(diallyldimethylammonium chloride) (PDADMAC) and poly(sodium 4-styrenesulfonate) (PSS) from solutions of different ionic strength [19–22]. This system is well-studied in literature and can be considered as a paradigm for the study of the multilayer behavior even though their practical applications are limited [16,19,23–25]. We have performed a study to analyze the effect of the ionic strength on the internal structure and composition of polyelectrolyte multilayers with a variety of techniques. Following this approach, we have contributed to solve some controversial aspects related to the role and distribution of the ions and water within the films, i.e., the internal composition of the films, as well as to the internal morphology of the films, i.e., the absence of stratification. Furthermore, the comparative study of multilayers as prepared and after drying has allowed us to deepen the understanding about the physicochemical foundations that govern the formation and properties of polyelectrolyte films.

## Experimental

### Chemicals

The poly(sodium 4-styrenesulfonate) (PSS) used had a molecular weight of 70 kDa. The poly(diallyldimethylammonium chloride) (PDADMAC) had a molecular weight in the range of 200–350 kDa. Both polymers were purchased from Sigma-Aldrich (Germany) and used without further purification. The ionic strength of the solutions was controlled by adding NaCl (Sigma-Aldrich, purity > 99.9%). The water used for all the experiments was of Milli-Q quality (Millipore RG model). All the experiments were done at (298.1 ± 1.0) K.

#### Layer-by-layer assembly

In a similar manner as described in [8], the multilayers were formed from polyelectrolyte solutions of different ionic strengths, *I*. Between the adsorption of successive layers, the multilayers were rinsed with the solvent used for preparing the polyelectrolyte solutions. The rinsing process removed the polymer chains that were not strongly adsorbed. Thus the fabrication of the films follows a typical adsorption sequence polycation–rinsing–polyanion–rinsing. All the adsorption steps were performed under static conditions, without any stirring in the adsorption cell.

For some of the studies performed, the drying of the multilayers was carried out between the rinsing and the deposition of the second polyelectrolyte layer following the above described procedure. For this purpose, the films were exposed to highly purified nitrogen flow after each adsorption–rinsing cycle.

#### Dissipative quartz crystal microbalance (QCM-D)

We have used a dissipative quartz-crystal microbalance (QCM-D) from KSV (Model QCM Z-500, Finland) for the study of the wet films, and an impedance/gain phase analyzer from Hewlett-Packard (HP4194A, U.S.A.) coupled to a QCM electrode for the study of the dry films. In similar manner as described in [19], the gold coated AT-cut quartz crystals were cleaned with piranha solution (70% H_2_SO_4_/30% H_2_O_2_) over a period of thirty minutes and then thoroughly rinsed with pure water. The characteristic frequency of the quartz crystal in vacuum was *f*_0_ ≈ 5 MHz. A self-assembled monolayer of sodium 3-mercapto-1-propanesulfonate was initially formed on the surface of the gold electrode of the quartz crystal, in order to obtain a charged substrate [19]. QCM-D provided the impedance spectra of the crystal for the fundamental resonance frequency and for its odd overtones, ν, up to 11 [26].

#### Ellipsometry

An imaging null-ellipsometer from Nanofilm (Model EP^3^, Germany) was used; all the experiments were carried out on a solid–liquid cell at a fixed angle of 60°. Silicon wafers (Siltronix, France) were used as the substrates. In order to obtain the ellipsometric thickness, *h*_op_, and the refractive index, *n*_f_, of the layers a four layer model has been used, as in a previous work [20]. From the results obtained from ellipsometry it was possible to calculate the mass adsorbed on the substrate, Г, using De Feijter’s equation [27],

[1]
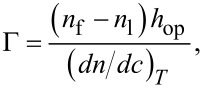


where *n*_f_ and *n*_l_, are the refractive index of the film and solvent, respectively. The *(dn*/*dc*)*_T_* values measured for PDADMAC and PSS are 0.213 and 0.178 cm^3^/g, respectively [20].

#### X-ray reflectivity

The reflectivity experiments were performed using silicon as substrates. X-ray experiments were made in a conventional diffractometer X’Pert Pro MRD from Panalytical (Netherlands). The analysis of X-ray reflectivity data was made using the software Package Parrat 32 from HMI (Berlin, Germany).

#### X-ray photoelectron spectroscopy

Surface chemical analysis of the samples was carried out by X-ray photoelectron spectroscopy (XPS) by using an ESCAPROBE P (Omnicron) spectrometer. The measurements were carried out with a Mg Kα (1253.6 eV) source operated at 150 W. The residual pressure was lower than 10^−7^ Pa during the collection of the spectra. The hemispherical analyzer EA 125 operated in constant analyzer energy mode and the pass energy was switched to 20 eV for transitions C 1s, Cl 2p, N 1s, Na 1s, O 1s and S 2p. Under these conditions the FWHM of the Ag 3d_5/2_ peak at 368.1 eV was 1.0 eV. Angle resolved spectra were collected at five sequentially increased electron emission angles to the normal of 10° from 0 to 40°, without modification of the source-to-detector configuration. This methodology provides information in depth equal to the cosine of the angle between the surface normal and the analysis direction. Data analysis of core level XPS spectra was conducted with Casa-XPS software, Relative sensitivity factors (RSF) employed: C 1s (1); Cl 2p (1.48); N 1s (1.77); Na 1s (7.99); O 1s (2.85); and S 2p (1.25).

#### Surface potential measurements

A Kelvin probe from Trek, Inc. (U.K.), located approximately 2 mm above the substrate, was used in order to measure the surface potential (Δ*V*) of the multilayer in the dry state after each cycle of deposition. The surface potential measurements are referenced to the value of Δ*V* of the bare solid–air interface.

#### Atomic force microscopy

AFM measurements were performed in air at room temperature using a Nanoscope III (Digital Instruments, USA) in the tapping mode. A silicon tip, model RTESP (Veeco Instrument Inc, USA), was used for the measurements. The AFM images were processed using the software WSxM from Nanotec Electronica [28].

## Results and Discussion

### Wet films vs dry films

**Figure 1:**
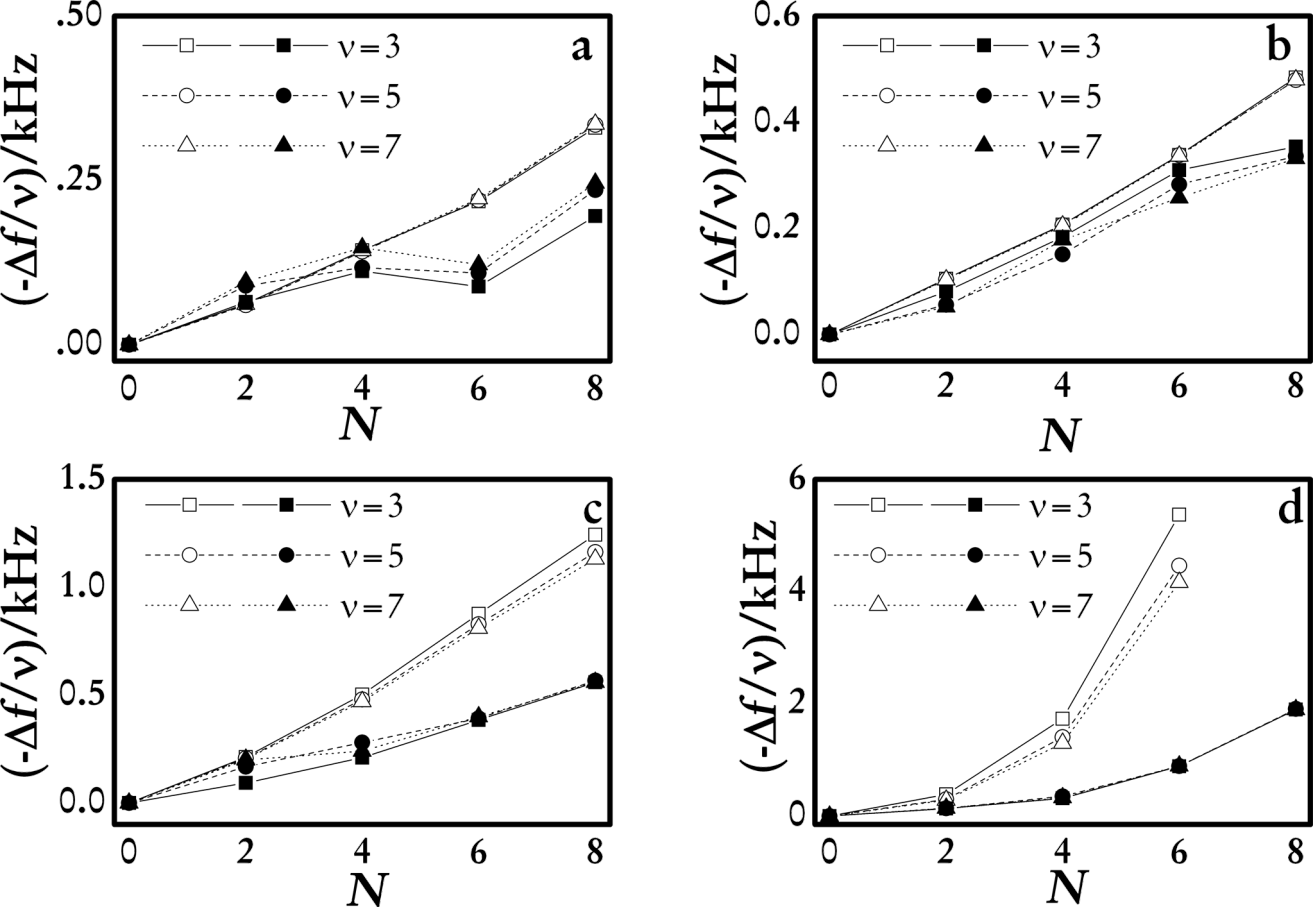
The reduced frequency of the quartz crystal for the different overtones measured (overtones ν = 3, 5 and 7) as a function of *N* for multilayers (PDADMAC + PSS)*_N_* formed from solutions with different ionic strength. (a) 0.05 M (b) 0.10 M (c) 0.30 M (d) 1.00 M. In all the plots: Wet multilayers (open symbols) and dry multilayers (solid symbols).

The growth of polyelectrolyte multilayers of (PDADMAC + PSS)*_N_* was followed by monitoring the frequency shift (Δ*f*) of the QCM-D normalized by the overtone number (ν), −Δ*f*/*ν*, as a function of the number of bilayers (*N*) [19,29]. It is well known that the adsorbed mass calculated using Sauerbrey’s equation underestimates the real mass of viscoelastic films [30–32]. Figure 1 shows the frequency shift as a function of *N* for wet and dry multilayers (PDADMAC + PSS)*_N_*. The differences between wet and dry multilayers are consequence of differences in the preparation method (see section “Layer-by-layer assembly”). The introduction of a drying step is expected to have a strong effect on the multilayer growth.

**Figure 2:**
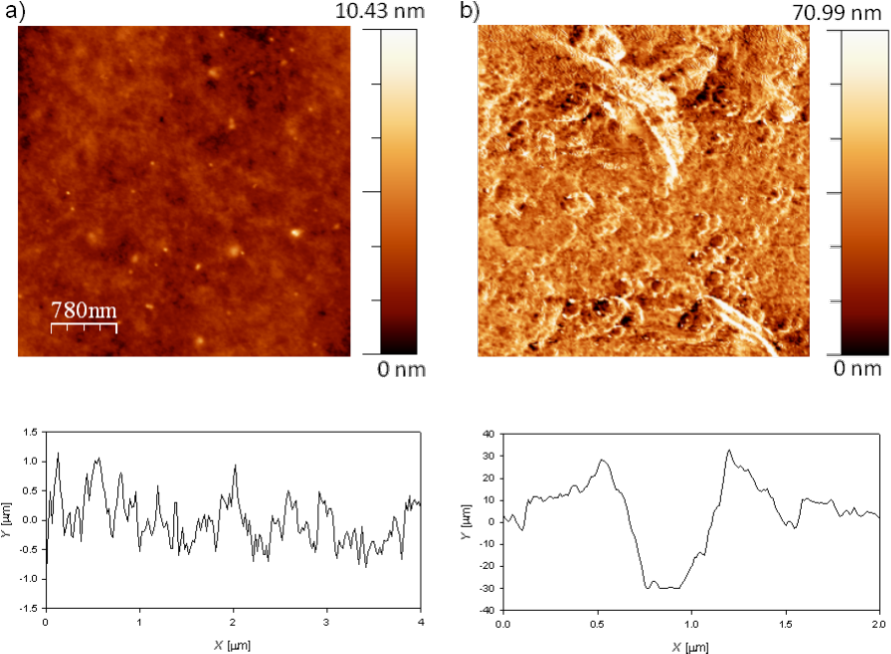
AFM images and height profiles taken along the diagonal of the images for multilayers (PDADMAC + PSS)*_N_* built using solutions with two different ionic strength. (a) 0.10 M (*N* = 15). (b) 1.00 M (*N* = 7).

From the results shown in Figure 1 it is possible to evaluate different aspects of the behavior of the multilayers related mainly to their growth. The adsorbed mass increases (higher decrease in the resonance frequency of the overtones) with the ionic strength, *I*, in both wet and dry films. This is explained considering the conformational changes of the polyelectrolyte chains due to the modification of the ionic strength [19]. In fact, the increase of *I* leads to a screening of the charge of the chains that leads to the change from an extended to a coiled conformation. Thus the number of molecules that can be adsorbed to coat the surface increases and consequently the surface coverage increases. Furthermore, the increase in *I* provokes the transition from a linear dependence of the mass on *N* to a non-linear one, in accordance with previous results [19,23–25]. High values of *I* lead to the adsorption of coiled chains, increasing the area available to the adsorption in the successive deposition cycles and consequently the growth dependence on *N* becomes supra-linear. This is also associated with an increase of roughness of the films (roughness data for different (PDADMAC + PSS)*_N_* are reported in Table 1) as it can also be seen in the AFM image analysis in Figure 2.

**Table 1:** Roughness for (PDADMAC + PSS)*_N_* multilayers at different ionic strengths (dry films).

c_NaCl_ [*M]*	*N*	roughness [nm]	technique

0.10	3	6 ± 2	X-ray reflectivity
	6	3.8 ± 0.5	AFM
	6	6 ± 1	X-ray reflectivity
	9	4 ± 1	X-ray reflectivity
	12	5 ± 1	X-ray reflectivity
	15	7.8 ± 0.5	AFM
	15	6 ± 2	X-ray reflectivity

0.50	12	12.1 ± 0.5	neutron reflectivity

1.00	7	17.8 ± 0.5	AFM

The AFM images (Figure 2) show a more inhomogeneous topology and a higher roughness for the film built using solutions with high ionic strength. Recent works have shown that the increase of roughness is deeply related to the non-linear growth [21–22,30–31,33]. But this influence of the increasing roughness on the transition between different growth mechanisms does not allow us to rule out the contributions associated with inter-diffusion of the polymers [10,34–35]. However, a quantitative discussion of the potential effect of the inter-diffusion to the multilayer growth on the basis of equilibrium results is difficult, and no additional discussion related to this aspect will be included.

It is worth mentioning that the growth trend, i.e., the dependency of the adsorbed mass on *N*, is not modified by the drying of the films. This allows us to suggest that the growth trend is determined exclusively by the specific interactions occurring in the system and the polymer conformation, without effects due to the hydration/swelling phenomena associated with the uptake/release of water.

Even though the *N* dependence of the adsorbed mass is not changed by the drying process, other aspects are strongly modified by the film drying, among them the most evident is the adsorbed mass (see Figure 1). Because the QCM-D detects both the polymer adsorbed and the hydration water, drying of the films reduces the adsorbed mass (lower resonance frequency shift). The drying process makes the polymer matrix shrink, which is critically related to the mechanical properties of the film. In fact, from the separation of the values of −Δ*f*/*ν* for the different overtones found in the QCM-D results [30–32], it is possible to predict the existence of modifications in the mechanical behavior of the films due to the drying process.

**Figure 3:**
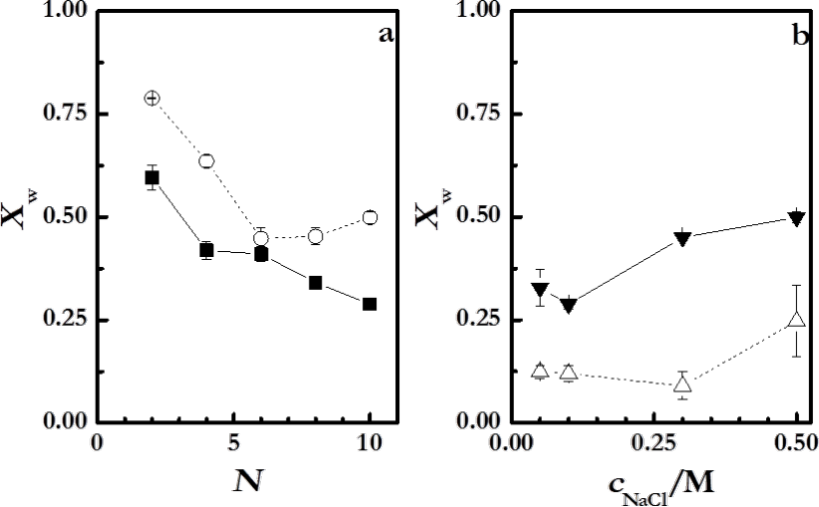
(a) Weight fraction of water as a function of *N*, obtained following the methodology proposed by Vöros [38], for (PDADMAC + PSS)*_N_* films formed at two different ionic strengths: 0.10 M (solid squares) and 0.50 M (open circles). (b) ) Weight fraction of water as a function of the ionic strength for (PDADMAC + PSS)*_N_* films: obtained through the methodology proposed by Vöros [38] (solid triangles) and through XPS (open triangles). The solid lines are to guide the eye.

The dependence of the normalized frequency on the number of the overtone allows us to make a qualitative discussion about the viscoelastic character of the layers [36]. The drying process leads to the collapse of the different overtones of the quartz crystal in a master curve. This is related to the transition from a viscoelastic behavior (lacked overlapping of the overtones) to a rigid one (Sauerbrey limit where the overtones define a master curve) [31]. Thus, it is possible to ascribe this change in the mechanical behavior of the layers to the release of water that leads to an increase of the ionic pairing and consequently to an increased rigidity of the multilayers in agreement with the results by Nolte and co-workers [37]. The presence of water induces a plastification of the film with the corresponding effect on the mechanical response of the multilayer. In addition, the increase of the ionic strength increases the viscoelastic character of the films, which is correlated to the formation of layers with more swollen chains [19]. It is expected that these swollen chains trap higher amounts of water, which leads to the most important plastifying effects [19]. We have calculated the water content using QCM-D and ellipsometry data following the method proposed by Vöros [19,38–41]. Figure 3 shows the water weight fraction, *X*_w_, for (PDADMAC + PSS)*_N_* films.

The water content decreases as *N* increases, which agrees with the behavior reported for other films [42–43]. This behavior is due to the fact that the first layers adsorbed form an inhomogeneous film, forming isolated island that coalesce as *N* increases [33,44–45]. This mechanism is also supported by the theoretical considerations based on the electrostatic interaction of charged objects onto opposite charged surfaces [46]. The results in Figure 3 point out that *X*_w_ slightly increases with ionic strength, as expected from the adsorption of more hydrated chains at higher values of ionic strength. Multilayers with high *N* present always average values of water content around the 20–60% of the total weight of the multilayer. Figure 3b shows that XPS technique leads to the same qualitative trend. Even though, the water content is not directly obtained from XPS measurements, it is possible to estimate it as

[2]
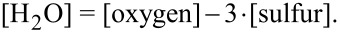


It is worth mentioning that the water content estimates from XPS refer to the water molecules that remain trapped in the multilayer after the drying process [47]. This allows for explaining the differences observed in the results obtained using the two procedures described above (see Figure 3b). The residual water that remains in the multilayer is more or less half of the quantity that exists under wet conditions, and it is related to the increase of the relative proportion of counterions in the multilayer with ionic strength as will be discussed in the following.

The swelling ratio of the films can be calculated following Schönhoff et al. [48] according to

**Figure 4:**
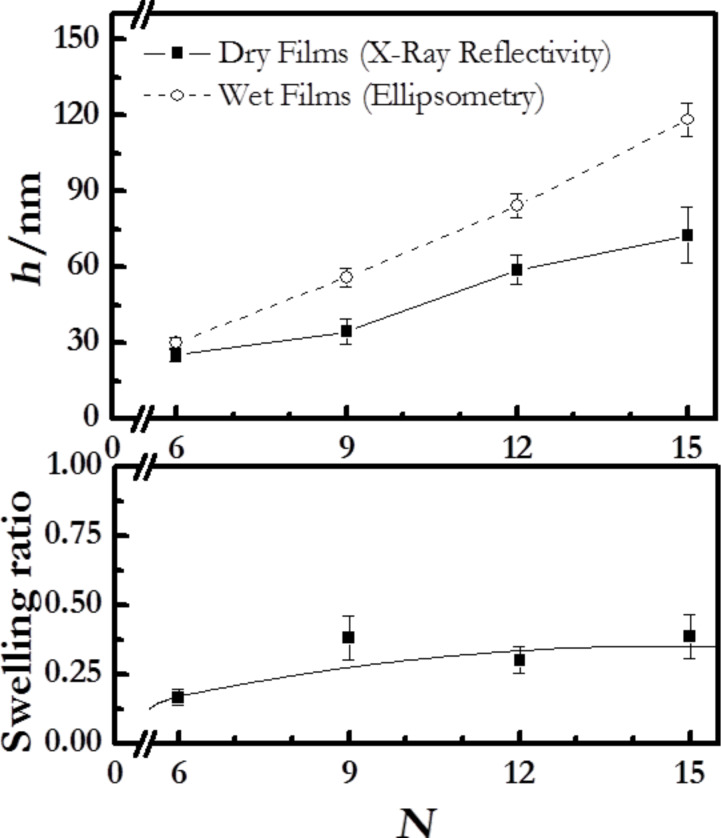
(a) Multilayer thickness of (PDADMAC + PSS)*_N_* films formed at an ionic strength of 0.10 M as a function of *N*, measured under wet conditions (ellipsometry) and dry conditions (X-ray reflectivity). (b) Swelling ratio as a function of *N* for the multilayers of part a. The lines are to guide the eye.

[3]
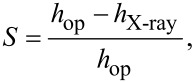


where *h*_op_ is the thickness calculated from ellipsometry for wet films, and *h*_X-ray_ the value obtained using X-ray reflectivity for dry multilayers. The results are shown in Figure 4.

For multilayers obtained at an ionic strength of 0.10 M, *S* is in the range of 20–35% and slightly increases with *N*, which is qualitatively similar to what was found by Dodoo and co-workes [49]. This is in agreement with a transition from an intrinsic compensation to an extrinsic one with the increase of *N* [50–51] that has a strong influence on the viscoelastic character of the film (Figure 1) [36] due to the reduction of the ionic cross-linking between chains in adjacent layers associated with the transition between intrinsic to extrinsic compensation. Table 2 reports the swelling ratio for multilayers formed at two different ionic strengths with *N* = 12.

**Table 2:** Thicknesses (ellipsometry (wet films), *h*_op_, and X-ray reflectivity (dry films), *h*_X-ray_) and swelling ratio, *S*, for (PDADMAC + PSS)*_N_* multilayers (*N* = 12) formed from solutions with two different ionic strengths.

c_NaCl_ [M]	wet films *h*_op_ [nm]	dry films *h*_X-ray_ [nm]	*S*

0.10	84 ± 5	59 ± 3	0.30 ± 0.05
0.50	316 ± 5	95 ± 13	0.67 ± 0.03

The degree of swelling of the multilayers can be related to the rigidification of the films upon drying (Figure 1). It is expected that films with low degree of swelling exhibit a strong ionic cross-linking under hydrated conditions with their rigidity almost unaffected during the drying process. On the other side, the films with the higher degree of swelling (under conditions of high ionic strength) are expected to exhibit a low level of ionic cross-linking, which leads to their rigidification upon dehydration. This is in accordance with the results obtained by Secrist and Nolte for spin-coated multilayers of (poly(allylamine) + poly(acrylic acid))*_N_* [52].

### Internal structure: evidence of a non-stratified system

X-ray photoelectron spectroscopy (XPS) provides valuable information about the surface chemistry of the samples. A method to provide depth profiles (with different penetration depths, *x*) is angle-resolved XPS. In this method the electron path through the solid, i.e., three times the inelastic mean free path, is related to the change of the emission angle, φ,

[4]
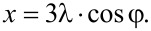


**Figure 5:**
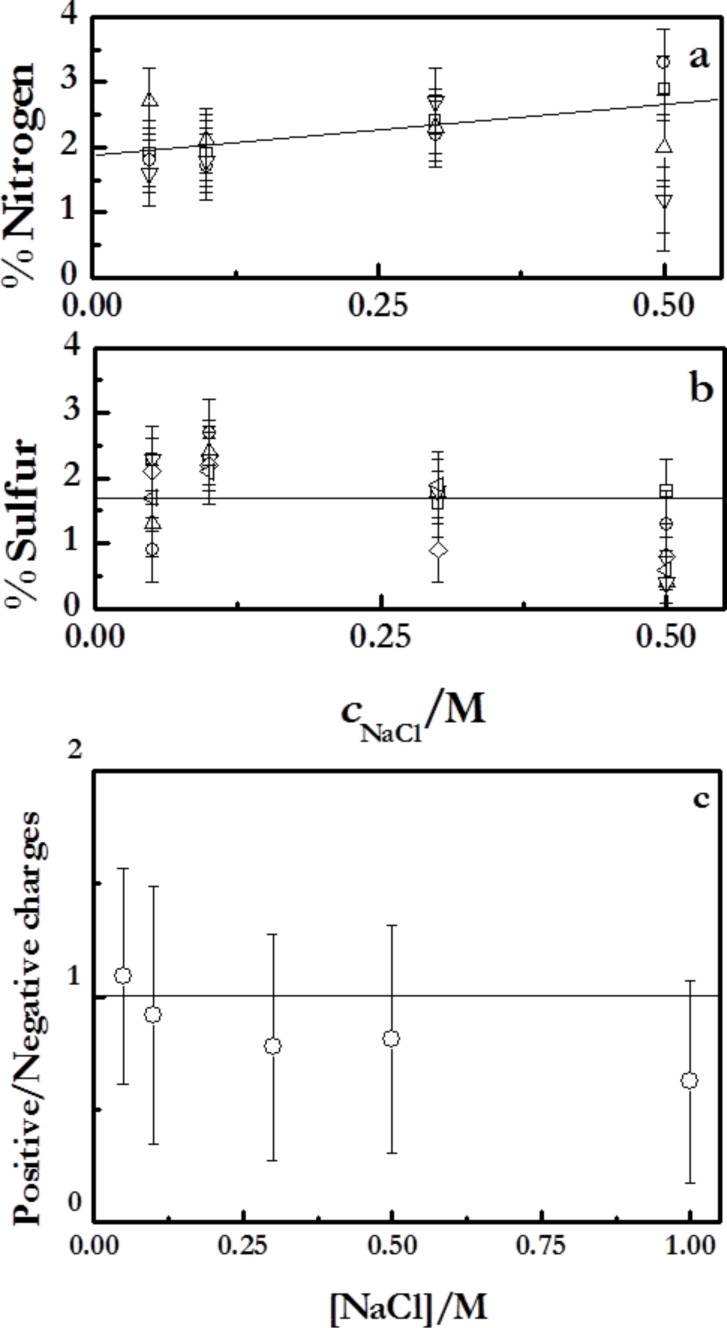
(a) Dependence on the salt concentration of the atomic fraction of nitrogen in (PDADMAC + PSS)*_N_* films, obtained by XPS measurement at different angles of electron emission: (squares) −40°, (circles) −30°, (upward triangles) −20°, (downward triangles) −10°, (diamonds) 0°, (leftward triangles) 10°. The solid lines are to guide the eye. (b) Dependence on the salt concentration of the atomic fraction of sulfur in (PDADMAC + PSS)*_N_* films, obtained by XPS measurement at different angles of electron emission: (squares) −40°, (circles) −30°, (upward triangles) −20°, (downward triangles) −10°, (diamonds) 0°, (leftward triangles) 10°. The solid lines are to guide the eye. (c): Dependence on the salt concentration for the ratio between positive and negative charges in (PDADMAC + PSS)*_N_* films, obtained by XPS measurement at normal angle of incidence and 0° emission angle. The solid line is to guide the eye.

At higher emission angles (with respect to the normal angle) a higher surface sensitivity is achieved. In order to obtain insights on the internal structure of the LbL films, we collected spectra at different emission angles while recording two different element representing each of the polymers, nitrogen for PDADMAC and sulfur for PSS. Thus, XPS can reveal information about a lamellar or disordered structure of the samples. Figure 5a and Figure 5b report the dependence of the element content of nitrogen and sulfur in the films on the ionic strength for different emission angles.

The atomic content remains almost constant with increasing ionic strength. Indeed, the atomic fractions of nitrogen and sulfur are both independent of the angle of incidence. This confirms the strong interdigitation of the successively adsorbed layers; PDADMAC and PSS form quasi-homogeneous mixed films. It is expected that this absence of stratification in the multilayer define the interactions within the film and the properties of the manufactured materials. Additional evidence of this absence of stratification can be obtained from the XPS measurements following the method proposed by Raposo and co-workers [47]. We have measured the ratio between the total content of positive (sodium and nitrogen) and negative charges (sulfur and chloride) in the multilayers. Values of this ratio close to unity indicate non-stratified films whereas values higher or lower than unity evidence the stratification of the multilayers [47]. Figure 5c shows the dependence of this ratio for (PDADMAC + PSS)*_N_* multilayers on the ionic strength. These results confirm the absence of stratification of the films, in agreement with previous studies based on reflectivity techniques (X-Ray and neutrons) [19,21–22], and contrast with the stratification found for [poly(allylamine) + PSS]*_N_* multilayers [17–18].

Moreover, a more detailed analysis of the XPS results in Figure 5a and Figure 5b provides additional insights in the adsorption of the different polymers in the multilayers. It is observed that the nitrogen content slightly increases with the ionic strength, while the content of sulfur remains constant, which means that the ionic strength affects the adsorption of PDADMAC but has a negligible effect on the adsorption of PSS. As it will discussed below this conclusion agrees with ellipsometry results.

**Figure 6:**
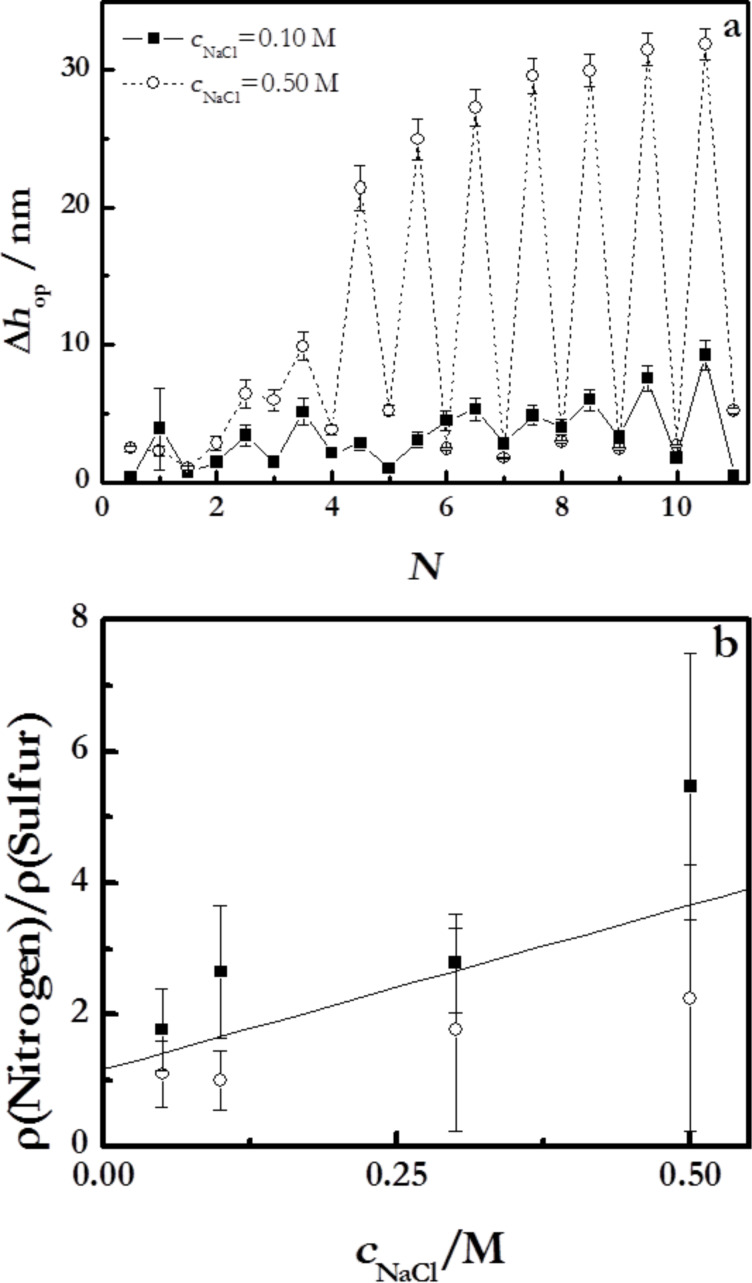
(a) Variation of thickness, obtained using ellipsometry for the adsorption of single layers, as a function of *N* for (PDADMAC + PSS)*_N_* films formed at two different ionic strengths: 0.10 M and 0.50 M. (b) Dependence on the salt concentration of the ratio between nitrogen and sulfur contents in (PDADMAC + PSS)*_N_* films obtained from ellipsometry (solid squares) measurements average over 12 bilayers and by XPS (open circles) measurements at 0°. The solid line is to guide the eye.

Ellipsometry is a technique that evaluates the adsorbed mass through the refractive index contrast between the adsorbed layers [53] and allows one to obtain the adsorbed mass of each layer. Figure 6a shows the thickness change, Δ*h*_op_, for the adsorption of each layer for wet multilayers (PDADMAC + PSS)*_N_* under different assembly conditions (linear and non-linear growth). They show a clear odd–even effect in the successive adsorption cycles [19,54]. These results confirm the dependence on the ionic strength of the adsorbed amount of PDADMAC discussed above (the change from 0.10 M to 0.50 M leads to a thickening of the PDADMAC layers by a factor of six), whereas the adsorption of PSS does not show any significant change. This reflects the importance of the assembly conditions in the control of the multilayers fabrication.

The ellipsometric thickness can be related to the number of polymeric chains by a simple modification of de Feijter’s equation (Equation 1) as we have discussed in a previous publication [42]. Following this approach it is possible to assume that the monomer surface density, ρ_monomer_, for each single layer can be obtained as

[5]
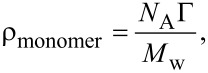


where *N*_A_ is the Avogadro constant, Г the surface concentration obtained by de Feijter’s equation (Equation 1) and *M*_w_ is the molecular weight of the monomers. Equation 5 quantifies the surface density of the marker atoms ρ(X) = ρ_monomer_ (X = nitrogen or sulfur). Considering this, we can define the ratio between the atomic contents of nitrogen and sulphur in the multilayer directly by the following expression,

[6]
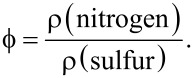


Figure 6b reports the average ratio between the atomic contents of nitrogen and sulphur in the multilayer obtained from ellipsometry. For this purpose, the average value obtained over twelve bilayers will be considered. The ratio obtained from XPS results obtained at normal emission angle is reported together to the average ratio calculated from Equation 6 based in ellipsometric measurements. The results obtained using both techniques show a good qualitative agreement. In both cases, an increasing trend is observed in the atomic ratio with the increase of the ionic strength.

### Chemical composition of the multilayers

**Figure 7:**
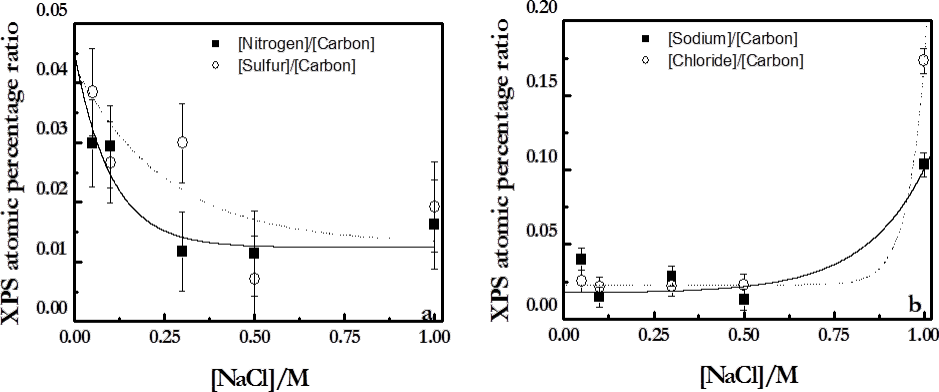
Atomic ratios obtained from XPS measurements at an angle of electron emission of 0°. (a) Ratios of [nitrogen]/[carbon] and [sulfur]/[carbon]. (b) Ratios [sodium]/[carbon] and [chloride]/[carbon]. The solid line is to guide the eye.

The XPS report allows one to perform a detailed chemical characterization of the multilayers. Figure 7 shows the chemical composition of the main components of the multilayers in relation to those of the carbon, since it is expected that the carbon content is not sensitive to *N*.

The contents of sulphur and nitrogen decrease with increasing ionic strength, whereas those of the counterions increase, in qualitative good agreement with the results of Raposo et al. [47] for multilayers of PSS + poly(*o*-methoxyaniline) emeraldine salt. This behavior is explained by the co-deposition of counterions with the polymer chains, which becomes more prominent as the ionic strength increases. This type of behavior is on the basis of a charge compensation mechanism that will be discussed below. The compensation mechanism is related to the relative proportion between the number of polymer chains defined by the nitrogen and sulfur contained in the multilayers and the number of counterions (sodium and chloride). Considering this fact, the simultaneous deposition of counterions and polymer chains from the bulk govern the ionic pairing between adjacent layers.

### Charge in polyelectrolyte multilayers

The charge of polyelectrolyte multilayers is one of the most critical aspects for the understanding of the physicochemical phenomena occurring in these soft systems. When we speak about the charge in polyelectrolyte multilayers, it is necessary to consider two different aspects that influence the film assembly: the charge inversion (overcompensation) that occurs during the deposition of the successive layers, and the charge compensation that ensures the neutrality of the supramolecular architecture.

**Figure 8:**
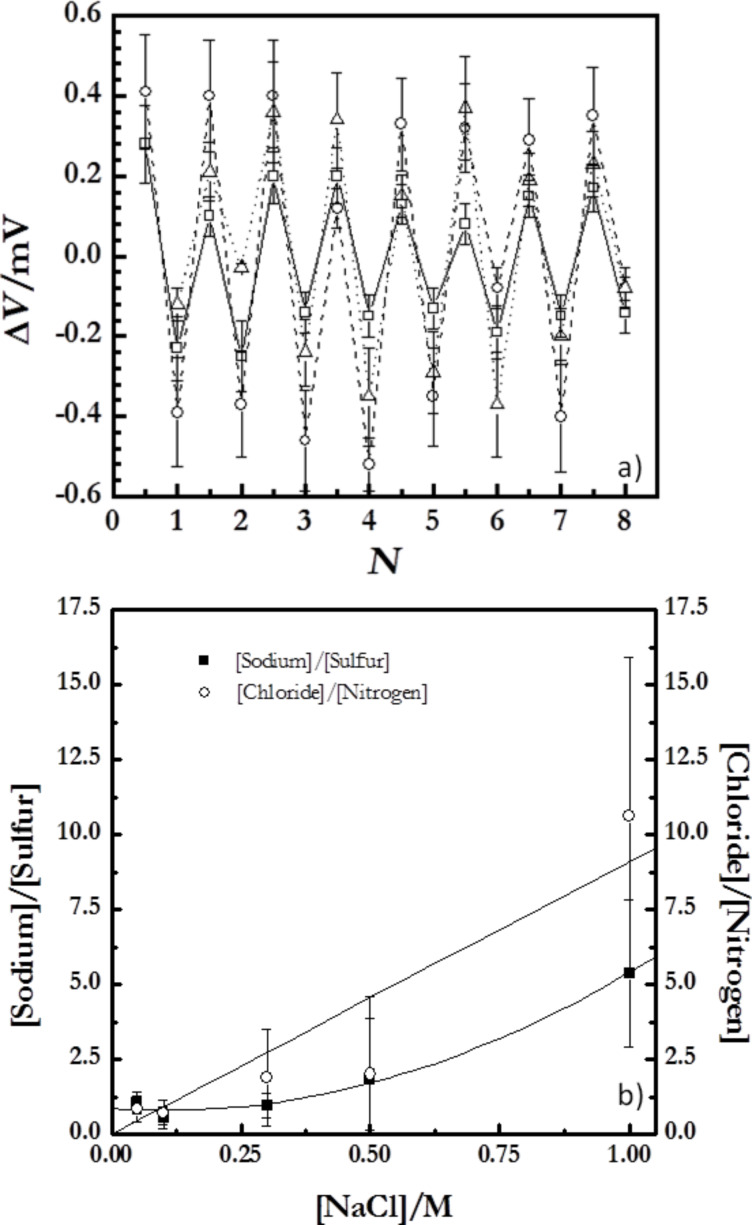
(a) Changes in the surface potential, Δ*V*, as a function of *N* for (PDADMAC + PSS)*_N_* films formed at different ionic strengths. The different symbols represent different ionic strengths: (open squares) 0 M, (open circles) 0.05 M, (open triangles) 1.00 M. (b) Ratios of [sodium]/[sulfur] and [chloride]/[nitrogen] as a function of the ionic strength obtained for dry multilayers from XPS spectra. The lines are to guide the eye.

The charge inversion or overcompensation has been traditionally considered as the main driving force for the assembly of polyelectrolyte films obtained by LbL methods [16,55]. In order to evaluate the charge inversion due to the sequential adsorption of layers with opposite charge, measurements of the changes of the surface potential, Δ*V*, have been performed (Figure 8a). The surface potential value changes between positive and negative values for the alternated adsorption of polycation and polyanion layers, respectively. Note that even the changes of the surface potential with *N* are similar to those expected for the ζ-potential; the absolute values measured by the Kelvin probe are referred directly to the potential on the surface whereas conventional measurements of ζ-potential are referred to an average charge within a larger area of the surface layer [56]. Figure 8a shows the surface potential of multilayers (PDADMAC + PSS)*_N_* adsorbed at different ionic strength as a function of *N*.

The Δ*V* values for the polyelectrolyte multilayers do not show any dependence on the ionic strength, which indicates the existence of a self-limited adsorption determined by the specific nature of the polyelectrolyte pair [22,57], i.e., the adsorption of a polyelectrolyte occurs until a certain degree of charge inversion is reached, independently of the assembly conditions. This is explained considering that the increase of the ionic strength reduces the effective charge density of the polyelectrolyte multilayers, thus the overcompensation threshold is reached for higher amounts of adsorbed polymer.

Despite the charge overcompensation, the multilayer must be neutral from a macroscopic point of view [15–16,58]. A quantitative evaluation of the compensation can be obtained from the ratio of monomers with positive and negative charge in adjacent layers as was described in [8,42]. This method showed the extrinsic compensation for the (PDADMAC + PSS)*_N_* system, independently of the ionic strength [19]. In addition, the content in PDADMAC is always higher than that of PSS as discussed above. This allows one to conclude that the content of chloride anions must be higher than that of the sodium cations. This latter is related to differences of the type of compensation implied in polycation and polyanion layers in agreement with the results by Lehaf and co-workers [59]. They found that PDADMAC-capped multilayers evidence a strongly extrinsic compensation whereas PSS-ended films are intrinsically compensated.

Moreover, the increase of the compensation ratio with the ionic strength is ascribable to the effect of entropic factors on the adsorption of the polyelectrolyte multilayers [16,19,58]. For low ionic strengths, the release of counterions strongly increases the entropic contribution to the adsorption process. This makes the charge compensation by the ionic pairing between polyelectrolytes in adjacent layers more favorable than the compensation through condensation of counterions. On the other side, the increase of the ionic strength reduces the importance of the entropic factor with the subsequent increases of the extrinsic compensation with counterions association to the films. Additional insights related to the charge compensation can be obtained using XPS [47,60–61]. Figure 8b reports the ratios of [sodium]/[sulfur] and [chloride]/[nitrogen].

Figure 8b shows that the role of the extrinsic compensation in PDADMAC layers is significantly enhanced with the increase in the ionic strength. This is associated to the strong screening effect of NaCl on the charge of PDADMAC. This implies that the amount of counterions associated with the PDADMAC layers increases with the ionic strength. On the opposite side, PSS layers show different behavior with the ratio that defines the degree of extrinsic compensation of PSS layers being almost independent on the ionic strength up to values higher than 0.5 M, where a slightly increase of the ratio of [sodium]/[sulfur] occurs. This is a further confirmation of the reduced effect of NaCl on the adsorption of PSS for low and moderate ionic strengths. The different trends found for the ratios of [sodium]/[sulfur] and [chloride]/[nitrogen] agree with the conclusion obtained from ellipsometry.

## Conclusion

The electrostatic self-assembly, using the LbL approach, of polyelectrolyte layers formed by PSS as polyanion and PDADMAC as polycation has been studied through different techniques that allowed for a better understanding of the multilayer internal composition and interactions. Different physicochemical aspects have been evaluated for this model system and it has been possible to conclude that the growth and properties of (PDADMAC + PSS)*_N_* films are mainly controlled by a complex interplay between three main parameters such as the hydration/swelling induced by the solvent, the charge compensation mechanism and the ionic pairing between polyelectrolytes in adjacent layers. The study of hydrated and dry films have demonstrated that the main physicochemical features of polyelectrolytes multilayers are similar independently of the hydration of the films with the water playing a key role in the swelling of the supramolecular architecture and adding mass to the hydrated films. The important contribution of the water as swelling agent of the films plays a central role for controlling the ionic cross-linking between adjacent layers, and consequently the mechanical properties of the films. The results have pointed out that swollen layers present always a most viscoelastic character than shrunk and dry films. The analysis of the structural aspects has pointed out the formation of intermixed layers of PDADMAC and PSS without evidences of stratification in the films.
